# Mammalian Glucose Transporter Activity Is Dependent upon Anionic and Conical Phospholipids*[Fn FN2]

**DOI:** 10.1074/jbc.M116.730168

**Published:** 2016-06-14

**Authors:** Richard C. Hresko, Thomas E. Kraft, Andrew Quigley, Elisabeth P. Carpenter, Paul W. Hruz

**Affiliations:** From the Departments of ‡ Pediatrics and; ¶ Cell Biology and Physiology, Washington University School of Medicine, St. Louis, Missouri 63110 and; the § Structural Genomics Consortium, University of Oxford, Oxford OX3 7DQ, United Kingdom

**Keywords:** glucose transport, glucose transporter type 4 (GLUT4), liposome, membrane transporter reconstitution, phospholipid, phospholipid vesicle, protein-lipid interaction, glucose transport kinetics, glucose transporter type 3 (GLUT3)

## Abstract

The regulated movement of glucose across mammalian cell membranes is mediated by facilitative glucose transporters (GLUTs) embedded in lipid bilayers. Despite the known importance of phospholipids in regulating protein structure and activity, the lipid-induced effects on the GLUTs remain poorly understood. We systematically examined the effects of physiologically relevant phospholipids on glucose transport in liposomes containing purified GLUT4 and GLUT3. The anionic phospholipids, phosphatidic acid, phosphatidylserine, phosphatidylglycerol, and phosphatidylinositol, were found to be essential for transporter function by activating it and stabilizing its structure. Conical lipids, phosphatidylethanolamine and diacylglycerol, enhanced transporter activity up to 3-fold in the presence of anionic phospholipids but did not stabilize protein structure. Kinetic analyses revealed that both lipids increase the *k*_cat_ of transport without changing the *K_m_* values. These results allowed us to elucidate the activation of GLUT by plasma membrane phospholipids and to extend the field of membrane protein-lipid interactions to the family of structurally and functionally related human solute carriers.

## Introduction

Membrane proteins are embedded in a lipid bilayer that covers a large percentage of their exposed surface. Lipid bilayers in typical human cells consist of hundreds of different lipid types that modulate cell signaling and protein function (1). Membrane lipid composition changes in several disease states and varies markedly over different cellular compartments. This influences the activity of transmembrane proteins by direct interactions with specific lipids or through changes in the biophysical properties of the membrane (*e.g.* fluidity, permeability, curvature, and lateral pressure) (2, 3). Each membrane protein interacts with several lipid molecules at any given time, yet understanding the functional and structural effects of these contacts has been previously limited. With the advent of several methodological advances in isolating and studying membrane proteins, the significance of individual lipid components has begun to emerge. Human and bacterial ion channels, aquaporin Z, LacY, and the nicotinic acetylcholine receptor, are among the growing list of membrane proteins that have been shown to require specific lipids like anionic phospholipids, phosphatidylethanolamine (PE),^6^ cholesterol, or signaling lipids like phosphatidylinositol bisphosphate for optimal activity or proper folding (4–11).

The solute carriers (SLCs) consist of over 400 family members in humans. A quarter of these are associated with disease, making them excellent targets for clinical research (12). Although highly important in nutrient uptake, drug transport, and waste removal, this class of proteins remains relatively understudied (12). Elucidation of the lipid requirement for activity of mammalian solute carriers remains of paramount interest for drug discovery and general understanding of transport mechanisms.

Members of the SLC2A family of facilitative hexose transporters are essential in regulating cellular metabolism by selectively transporting glucose down a concentration gradient without the use of energy. To date, 14 distinct human SLC2A isoforms (GLUTs) have been identified (13), and for several of these proteins the cellular function in health and disease has been established. Regulation of the insulin-responsive facilitative glucose transporter GLUT4 (SLC2A4), which is primarily responsible for mediating peripheral glucose disposal in insulin-sensitive tissues such as skeletal and cardiac muscle as well as adipose tissue (14), has been intensively studied in relation to the pathophysiology of type 2 diabetes mellitus (15). Under basal conditions, the majority of GLUT4 remains sequestered within the cytosol in specialized membrane vesicles. Upon stimulation by insulin, a complex signaling cascade is initiated that results in the translocation of GLUT4 to the cell surface (16). In contrast, GLUT3 (SLC2A3), which has been classically defined as the neuronal glucose transporter due to the high level of expression and initial characterization in nervous tissue (17), is constitutively present on the cell plasma membrane. GLUT3 is also highly expressed in other tissues with high energy needs, including sperm, embryonic tissue, and leukocytes (18). Despite the longstanding recognition of an association between changes in membrane lipid composition and glucose transport in human disease (19, 20), the role of specific phospholipids on the functional activity of individual GLUT isoforms remains elusive. Although initial studies on the influence of lipids on the activity of GLUT1 isolated from erythrocyte membranes were performed nearly 30 years ago (21), until recently it was not possible to isolate and reconstitute other GLUT isoforms in a functional form to allow comprehensive evaluation of membrane lipids, alone or in combination, at physiologically relevant levels.

Here, for the first time we demonstrate that the glucose transport activity of GLUT4 and GLUT3 is controlled by the membrane phospholipid composition. Based on biochemical data and on crystal structures of GLUT3 and the close homologues GLUT1 and GLUT5 in multiple conformations, the currently accepted mechanism of action for these transporters is defined by interconversions between inward- and outward-facing conformations via a rocker-switch motion, governing the transport of solutes over the membrane barrier (22–24). Our findings expand the current model by introducing the modulation of transporter activity by specific phospholipids, thereby highlighting the importance of two classes of phospholipids that might regulate the activity of other members of the SLC family.

## Results

### 

#### 

##### GLUT4 and GLUT3 Show Specific and Saturable Uptake of Glucose in Liposomes of Defined Phospholipid Composition

**FIGURE 1.**
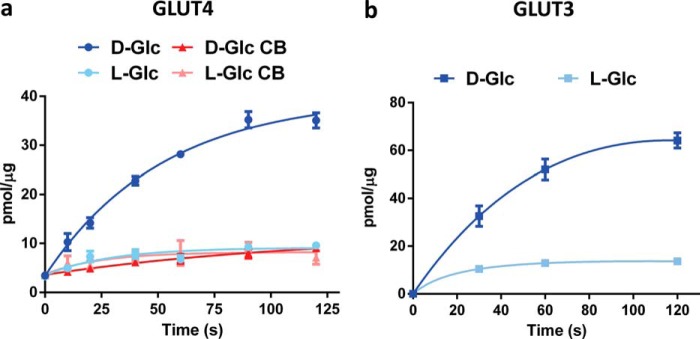
**Liposomes reconstituted with GLUT4 or GLUT3 transport d-glucose with high specificity over l-glucose.** *a,* time-dependent uptake of specific d-[^3^H]glucose and nonspecific l-[^3^H]glucose into GLUT4-containing PC/POPA/POPE (70:15:15) liposomes in the presence or absence of 20 μm GLUT-specific inhibitor cytochalasin B (*CB*). Data are the mean ± S.E. of three independent experiments. *b,* time-dependent uptake of specific d-[^3^H]glucose and nonspecific l-[^3^H]glucose into GLUT3-containing PC/POPA/POPE (70:15:15) liposomes. Data are expressed as mean ± S.E. of three independent experiments. *Lines* depicted in *a* and *b* are non-linear fits of the data to guide the readers' eyes.

**FIGURE 2.**
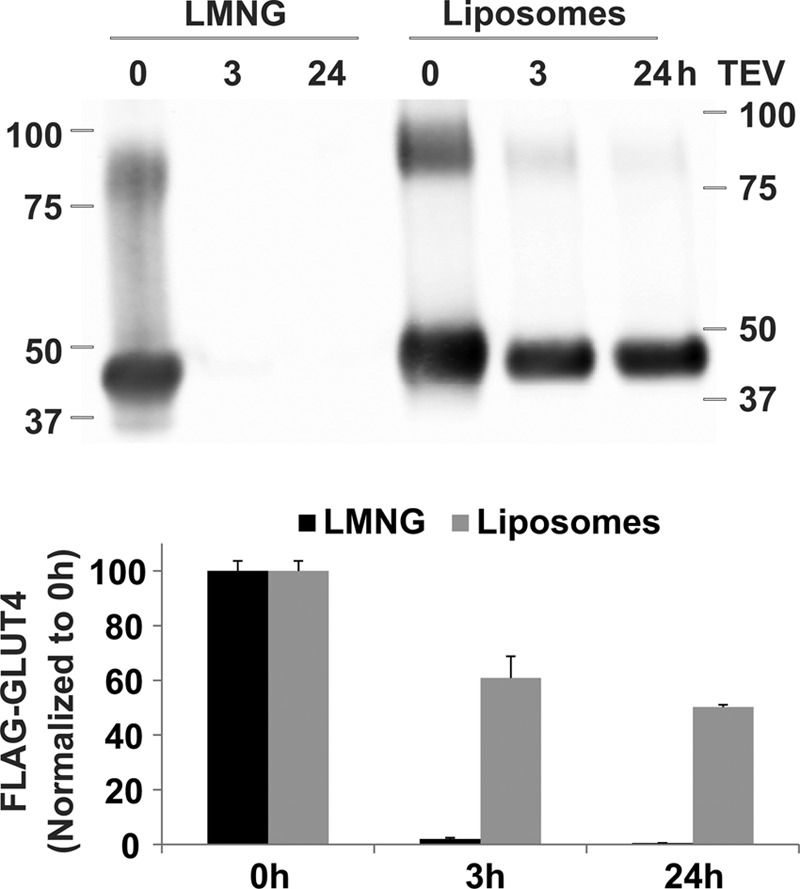
**GLUT4 is randomly inserted into liposomes.** Western blotting analysis of FLAG-GLUT4 using anti-FLAG antibody before and after TEV protease cleavage of an engineered N-terminal FLAG tag. GLUT4 cleavage was carried out in LMNG detergent micelles or in 70% egg PC, 15% POPA, 15% POPE liposomes for 0, 3, and 24 h. FLAG-GLUT4 (sum of both monomer and dimer) was quantified using an Odyssey Infrared Imaging System.

To study the effect of different phospholipids at specific concentrations on the activity of GLUT4 and GLUT3, we developed an assay system allowing us to measure uptake of radiolabeled glucose into liposomes that contained purified transporter embedded in a tightly controlled lipid environment. Both transporters showed saturable uptake over seconds to minutes of their primary substrate d-glucose over the non-transportable l-glucose in liposomes containing 70% egg phosphatidylcholine (egg PC), 15% phosphatidic acid (PA), and 15% PE (mol/mol) (Fig. 1). Uptake was normalized to the amount of transporter in each uptake assay as described under “Experimental Procedures.” Transport could be completely abolished using the GLUT-specific inhibitor cytochalasin B (Fig. 1*a*). As expected for random insertion, the transporter incorporated into liposomes with roughly half in an outward-facing and half in an inward-facing orientation. Evidence for this conclusion was obtained from Western blotting analysis of the transporter using anti-FLAG antibody before and after tobacco etch virus (TEV) protease cleavage of an engineered N-terminal FLAG tag (Fig. 2). In detergent micelles, the protease has access to both faces of the transporter, although in liposomes, it can only access transporters that orient with their N terminus facing outward. We determined cleavage of the FLAG tag is ∼100% in micelles, whereas only ∼50% of all transporters in liposomes are cleaved by TEV protease. Interestingly, the immunoblot revealed that a fraction of GLUT4 in both detergent and liposomes ran as a dimer when tagged with FLAG but as a monomer when cleaved. All subsequent liposome experiments were carried out using tag-cleaved GLUT4 or GLUT3.

##### Anionic Phospholipids Stabilize GLUT4 and Are Required for Activity of GLUT4 and GLUT3

**FIGURE 3.**
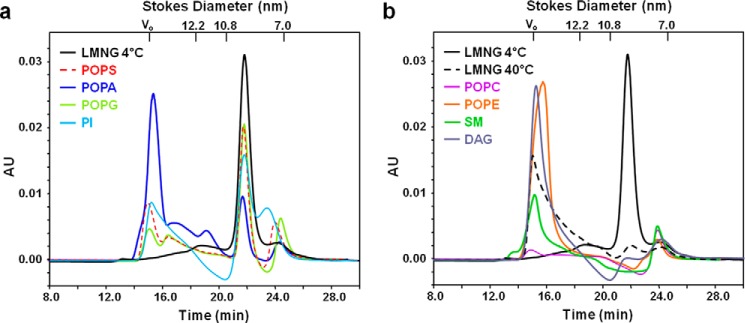
**Protection of GLUT4 by anionic phospholipids from heat-induced destabilization.** SEC elution profiles of GLUT4 after heating in the presence or absence of several anionic (*a*) and zwitterionic (*b*) lipids (0.02% w/v) as indicated by the color key. A profile of unheated GLUT4 in LMNG micelles (LMNG 4 °C) is shown for comparison. Protein absorption was monitored at 280 nm at a flow rate of 0.55 ml/min. Results are representative of two independent experiments. Apoferritin, β-amylase, bovine serum albumin (Stokes diameters, 12.2, 10.8, and 7 nm, respectively), and blue dextran (void volume, *V*_0_) were used as molecular mass standards for the SEC column.

We hypothesized that similar to several ion channels and receptor proteins, mammalian transporter function is regulated by its lipid environment. Several studies have shown that specific binding of annular lipids to various membrane proteins stabilize their structure and modulate their function (25–27). For example, Laganowsky *et al.* (26) demonstrated the stabilizing effect of lipids directly binding to several membrane proteins by preventing gas-phase unfolding in ion-mobility mass spectrometry experiments at increasing voltages. Similar to this approach, we devised a simple method to efficiently identify lipids that stabilize the transporter. Microgram amounts of lauryl maltose neopentyl glycol (LMNG) detergent-purified monomeric GLUT4 were incubated in the presence or absence of various lipids followed by thermal destabilization. The ability of specific lipids to stabilize the folded protein was assessed via size exclusion chromatography (SEC). We previously established that a single symmetrical peak in the SEC elution profile corresponds to monomeric, properly folded, active GLUT4, whereas multiple asymmetric peaks that elute earlier are an indicator of a heterogeneous population of destabilized, unfolded, or aggregated protein (28). Monomeric GLUT4 eluted at 21.5 min (Fig. 3), near the predicted elution time of monomeric GLUT4 LMNG micelles (∼175 kDa = LMNG micelle (120 kDa) + GLUT4 (55 kDa)). The molecular mass standards of β-amylase (200 kDa) and bovine serum albumin (66 kDa) eluted at 20.6 and 24.7 min, respectively. In the presence of anionic phospholipids, a monomeric peak of folded GLUT4, comparable with unheated folded GLUT4 in LMNG micelles, was retained on the SEC profile (Fig. 3*a*), whereas zwitterionic and non-bilayer phospholipids did not stabilize the transporter, similar to heated GLUT4 in LMNG micelles (Fig. 3*b*).

**FIGURE 4.**
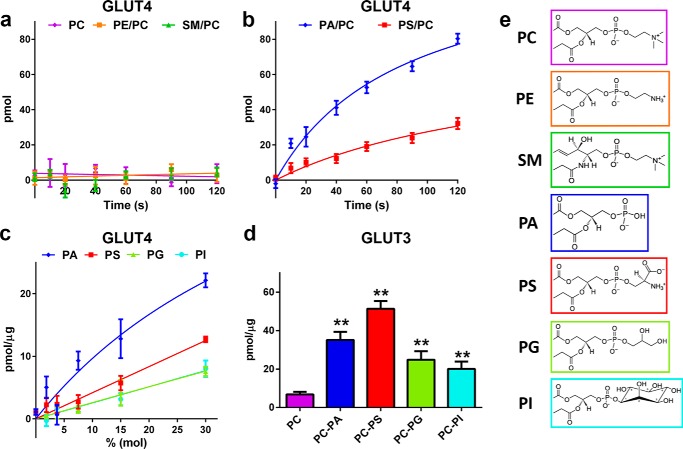
**Anionic phospholipids are required for GLUT4- and GLUT3-mediated d-glucose uptake.** *a* and *b,* time-dependent specific uptake of d-[^3^H]glucose into GLUT4-containing PC liposomes in the presence or absence of 15% (mol/mol) zwitterionic lipids (*a*) and anionic phospholipids (*b*). Data are the mean ± S.E. of three independent experiments. *c,* specific uptake of d-[^3^H]glucose into GLUT4-containing PC liposomes with increasing concentrations of anionic phospholipids. Uptake data were normalized to GLUT4 concentration as described under “Experimental Procedures.” Uptake data (40 s) are the mean ± S.E. of three independent experiments. *d,* specific uptake of d-[^3^H]glucose into GLUT3-containing PC liposomes in the presence or absence of 15% (mol/mol) anionic phospholipids. Uptake data were normalized to GLUT3 concentrations as described under “Experimental Procedures.” Uptake data (40 s) are the mean ± S.E. of four independent experiments. *Asterisks* denote statistically significant differences (**, *p* ≤ 0.01) compared with PC control as determine by one-way ANOVA plus *a posteriori* Holm-Sidak test. *e,* chemical structure of each of the lipid headgroups are colored-coded for comparison. *Lines* depicted in *a–c* are non-linear fits of the data to guide the readers' eyes.

The observation that only anionic phospholipids stabilize the transporter structure prompts the question as to whether these lipids also modulate the function of GLUT4. We assessed the activity of the transporter in liposomes containing 100% egg PC or egg PC liposomes containing a second lipid at an 85:15, molar ratio. All glucose uptake data described in this study are presented as specific d-glucose uptake, calculated by subtracting nonspecific l-[^3^H]glucose from d-[^3^H]glucose uptake, to take into account the possibility of membrane leakage. No specific GLUT4-mediated uptake was observed in the zwitterionic phospholipids PC, PE, or sphingomyelin (SM) (Fig. 4*a*). In contrast, the addition of the anionic phospholipids, PA or phosphatidylserine (PS), activated the transporter and resulted in specific d-glucose uptake (Fig. 4*b*). These results indicate a requirement for anionic phospholipids for transport activity and confirm our SEC results. To study the effect of the major naturally occurring anionic phospholipids in eukaryotic membranes in detail, we titrated palmitoyloleoyl phosphatidic acid (POPA), palmitoyloleoyl phosphatidylserine, palmitoyloleoyl phosphatidylglycerol, and phosphatidylinositol (PI) into PC liposomes and measured GLUT4-mediated uptake of d-glucose (Fig. 4*c*). All four anionic phospholipids led to a dose-dependent activation of GLUT4 with PA exhibiting the strongest effect, PS the second, followed by PI and phosphatidylglycerol. Anionic phospholipids also activated GLUT3 but with important differences (Fig. 4*d*). Unlike GLUT4, GLUT3 possessed measurable, albeit low, transport activity in the absence of anionic phospholipids. The addition of anionic phospholipids greatly enhanced GLUT3 activity, with PS having the most potent effect, followed by PA, and then phosphatidylglycerol and PI. The SEC and transport results suggest that anionic phospholipids interact directly with the transporter to stabilize its structure and are required for optimal GLUT4 and GLUT3 activity.

##### Conical Lipids Stimulate Glucose Uptake in Liposomes That Contain Anionic Phospholipid

**FIGURE 5.**
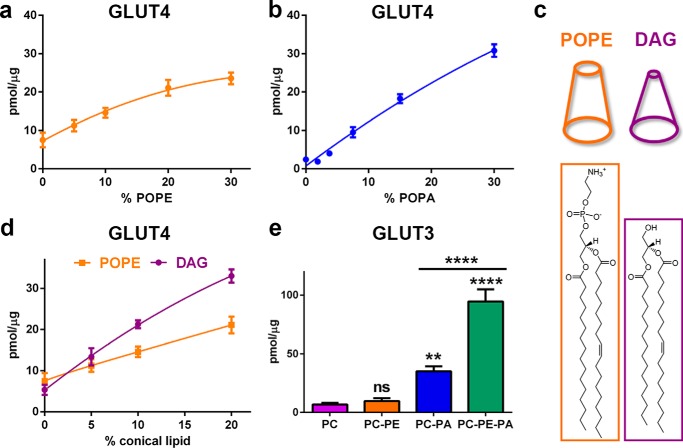
**Conical lipids stimulate GLUT4- and GLUT3-mediated glucose uptake.** *a,* specific uptake of d-[^3^H]glucose into GLUT4-containing PC liposomes in the presence of 10% (mol/mol) POPA with increasing concentrations of POPE. Normalized uptake data (40 s) are the mean ± S.E. of five independent experiments. *b,* specific uptake of d-[^3^H]glucose into GLUT4-containing PC liposomes in the presence of 30% (mol/mol) POPE with increasing concentrations of POPA. Normalized uptake data (40 s) are the mean ± S.E. of five independent experiments. *c,* chemical structures and overall shape of POPE and DAG. *d,* specific uptake of d-[^3^H]glucose into GLUT4-containing PC liposomes in the presence of 10% (mol/mol) POPA with increasing concentrations of DAG. POPE titration results are shown for comparison. Normalized uptake data (40 s) are the mean ± S.E. of five independent experiments. *e,* specific uptake of d-[^3^H]glucose into GLUT3-containing PC liposomes in the presence or absence of 15% (mol/mol) POPA and/or 15% (mol/mol) POPE. Uptake data (40 s) are the mean ± S.E. of five independent experiments. *Asterisks* denote statistically significant difference (*ns*, *p* > 0.05; **, *p* ≤ 0.01; ****, *p* ≤ 0.0001) compared with PC control (unless otherwise indicated) as determine by one-way ANOVA plus *a posteriori* Holm-Sidak test. Uptake data were normalized to GLUT4 or GLUT3 protein concentration as described under “Experimental Procedures.” *Lines* depicted in *a, b,* and *d* are non-linear fits of the data to guide the readers' eyes.

Nonbilayer lipids like palmitoyloleoyl phosphatidylethanolamine (POPE) have been shown to increase the activity of several membrane proteins in the presence of anionic phospholipids (6, 29–31). To assess the effects of POPE on GLUT4 activity, we measured glucose transport in liposomes containing 10% POPA in egg PC, titrated with increasing amounts of POPE. POPE significantly increased glucose uptake reaching a maximum effect of 3-fold at 30% POPE (Fig. 5*a*). We next determined the effect of anionic phospholipids on GLUT4 activity in the presence of POPE. Glucose transport measured in liposomes containing 30% POPE in egg PC were titrated with POPA. As expected, POPA stimulated glucose uptake in a concentration-dependent manner, and no specific uptake was observed in 30% POPE in the absence of anionic phospholipid (Fig. 5*b*). Unlike the other predominant mammalian zwitterionic lipids PC or SM, POPE is conically shaped with a small headgroup and one unsaturated acyl chain that occupies a larger volume than saturated chains. To determine whether the activating effect of POPE on GLUT4 is mediated by a specific interaction of the lipid to the transporter, as suggested for anionic phospholipids, or by altering the biophysical properties of the lipid bilayer, we compared the effect of the non-bilayer lipid palmitoyloleoyl glycerol (DAG) to POPE. Both lipids share the same aliphatic tails but significantly differ in their headgroup with DAG completely lacking the phosphate group and therefore exhibiting an even stronger conical shape than POPE (Fig. 5*c*). In the presence of DAG, GLUT4 activity was increased in a dose-dependent manner, similar to POPE (Fig. 5*d*). DAG showed a more pronounced effect on transporter activity with a maximal activation of >2-fold higher than POPE, correlating with its stronger conical shape. As the amount of DAG in the plasma membrane (PM) is a small fraction of PE (32), it is unlikely that this lipid has a physiological role in activating GLUT4. Nevertheless, this experiment demonstrates that POPE and DAG activate GLUT4 not through a headgroup specific effect but through their conical shape via changes in the biophysical properties of the lipid bilayer (1, 3, 33). This effect was also observed for GLUT3 (Fig. 5*e*). The addition of 15% conical lipids (POPE) increased transporter activity by 2.7-fold in the presence of anionic phospholipids. Similar to GLUT4, conical lipids did not significantly increase transporter activity of GLUT3 in the absence of anionic phospholipids. These results establish that conical lipids are necessary but not sufficient for optimal GLUT4 and GLUT3 transport activity.

##### Anionic and Conical Lipid Increase Transporter Activity in Liposomes with Physiological Lipid Concentrations

**FIGURE 6.**
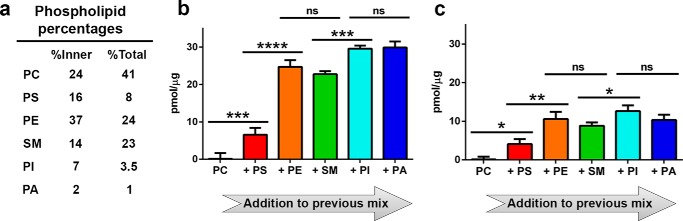
**Anionic and conical lipid effects on GLUT4-mediated glucose uptake are observed in liposomes with a lipid composition comparable with that of the PM.** *a,* phospholipid composition of an idealized mammalian PM (32). *b* and *c,* specific uptake of d-[^3^H]glucose into GLUT4-containing liposomes with lipid ratios reflecting the inner leaflet (*b*) or total PM (*c*) composition. From *left to right,* each *bar* represents an experiment where an additional lipid was added to the previous lipid mix (supplemental Tables 1 and 2). Uptake data were normalized to GLUT4 concentration as described under “Experimental Procedures.” Uptake data (40 s) are the mean ± S.E. of five independent experiments. *Asterisks* denote statistically significant difference (*ns*, *p* > 0.05; *, *p* ≤ 0.05; **, *p* ≤ 0.01; ***, *p* ≤ 0.001; ****, *p* ≤ 0.0001) as determined by one-way ANOVA plus *a posteriori* Holm-Sidak test. Comparison of +PE to +PI mixtures were significant in *b* (*p* < 0.02) but not in *c*.

Like DAG, the levels of PA in mammalian PMs are quite low (∼1%), and therefore the more abundant anionic phospholipids PS and PI most likely contribute more significantly in terms of enhancing normal glucose transport activity. To determine whether the anionic and conical lipid effects are also observed in a more physiological environment, GLUT4 activity was measured in liposomes with a phospholipid composition comparable with that of a mammalian PM (Fig. 6*a*) (32). Cell plasma membranes show trans-bilayer lipid asymmetry with anionic phospholipids being found exclusively and conical lipids preferentially on the cytoplasmic face (inner leaflet), a feature that distinguishes eukaryotic from prokaryotic membranes (1). Because of the technical challenge of generating asymmetric vesicles that recapitulate native mammalian cell membranes, characterization of the major phospholipids was carried out in liposomes composed of lipid levels found in either the PM inner leaflet (Fig. 6*b*) or the total PM membrane (Fig. 6*c*). To assess the contribution of each individual phospholipid on glucose transport, we determined transporter activity in liposomes after subsequent additions of lipids to the previous mix, starting with egg PC. From left to right, each bar represents an experiment in which an additional lipid was added to the previous lipid mix thereby reducing the molar amount of egg PC. The precise lipid compositions for each experiment can be found in supplemental Tables 1 and 2. As expected, in the presence of only the zwitterionic cylindrical lipid egg PC, no specific uptake was detected, although the addition of the anionic phospholipid PS significantly increased d-glucose uptake above background. Uptake was dramatically enhanced upon further addition of the conical lipid PE. Adding the other zwitterionic lipid SM to the system had no effect on glucose uptake, although the addition of physiological levels of PI to the lipid mixture containing PC, PS, PE, and SM further increased uptake. PA addition had no measurable effect on transport suggesting that the low level addition of PA was below the detection level for this assay. As anticipated, liposomes resembling the inner leaflet lead to higher GLUT4 activity due to the higher concentrations of anionic and conical lipids compared with liposomes composed of lipid compositions that approximate total PM.

##### Anionic and Conical Lipids Increase the Turnover Number of GLUT4 and GLUT3 but Not Their Substrate Binding Affinity

**FIGURE 7.**
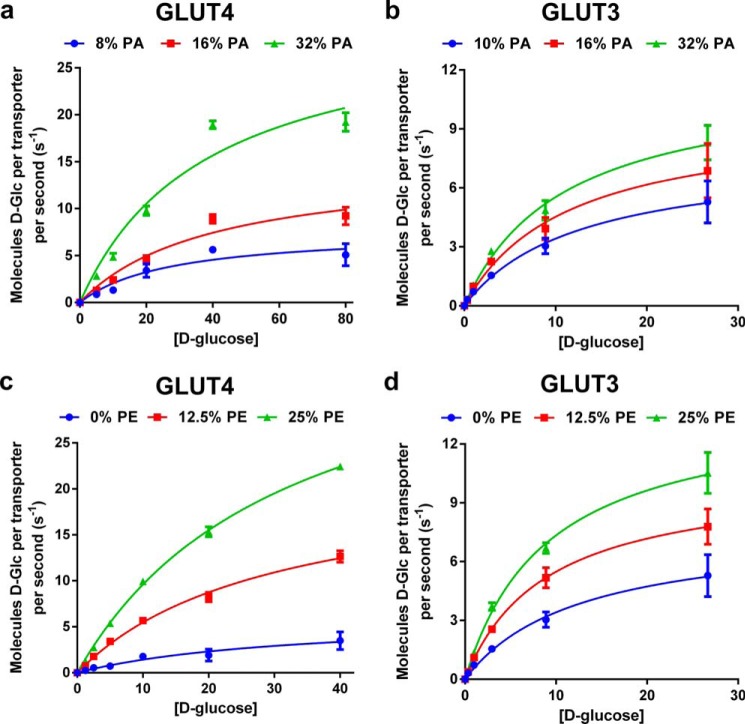
**Influence of anionic and conical lipids on transporter kinetics for GLUT4 and GLUT3.** *a* and *b,* d-glucose concentration (millimolar) dependence of the transport rate of GLUT4 (*a*) and GLUT3 (*b*) reconstituted in PC liposomes containing several concentrations of POPA. Normalized data are expressed as mean ± S.E. of four independent experiments. *c* and *d,* d-glucose concentration (millimolar) dependence of the transport rate of GLUT4 (*c*) and GLUT3 (*d*) reconstituted in 10% POPA-PC liposomes containing several concentrations of POPE. Normalized data are expressed as mean ± S.E. of four independent experiments. *Lines* depicted in this figure are from non-linear regression analysis using Michaelis-Menten enzyme kinetics.

**FIGURE 8.**
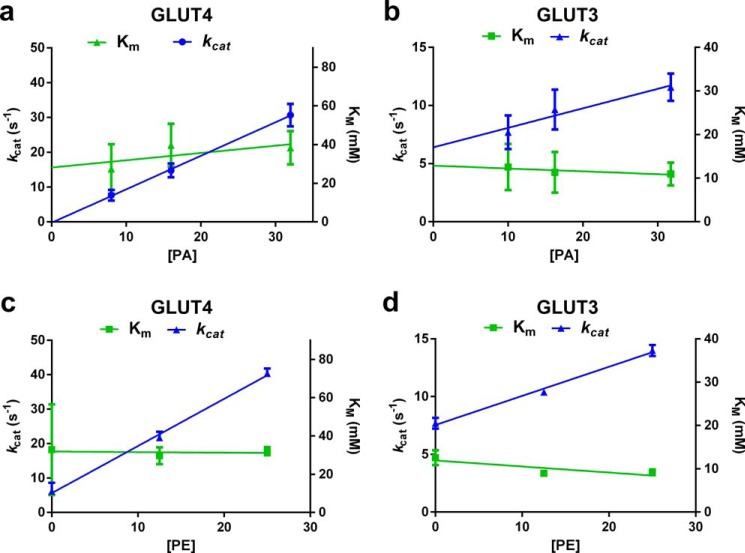
**Anionic and conical lipids increase the *k*_cat_ but have no effect on the *K_m_* values of both GLUT4 and GLUT3.** *a* and *b,* POPA concentration (mol %)-dependent change of the kinetic parameters *k*_cat_ and *K_m_* for GLUT4 (*a*) and GLUT3 (*b*). Data are expressed as mean ± S.E. of four independent experiments. *c* and *d,* POPE concentration (mol %)-dependent change of the kinetic parameters *k*_cat_ and *K_m_* for GLUT4 (*c*) and GLUT3 (*d*). Data are expressed as mean ± S.E. of four independent experiments. Kinetic parameters were determined from data shown in Fig. 7 by non-linear regression analysis and fitted using linear regression analysis.

**FIGURE 9.**
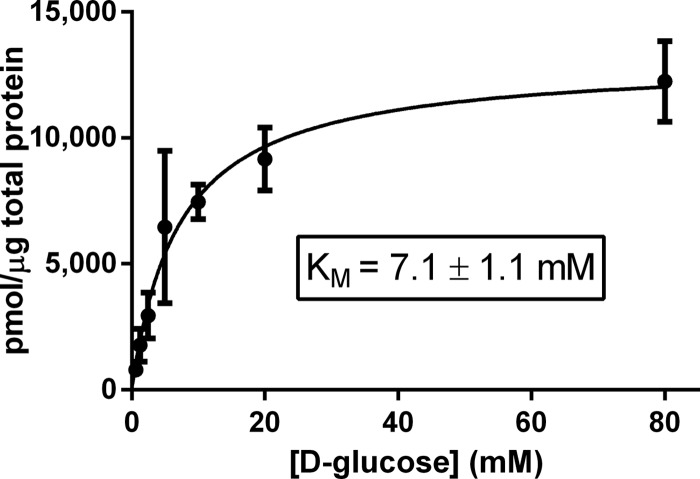
**d-Glucose concentration-dependent transport rate of GLUT4 in HEK293 cells.** Normalized data are expressed as mean ± S.E. of four independent experiments. Michaelis constant *K_m_* was determined by non-linear regression analysis using GraphPad Prism 6.0.

We used kinetic analysis to determine the mechanism of lipid-induced changes of glucose transporter function. The rate of zero-trans uptake of radiolabeled d-glucose into transporter-containing liposomes corrected for nonspecific uptake and normalized for the amount of transporter per assay was determined at increasing substrate concentrations with different amounts of POPA added to PC liposomes (Fig. 7, *a* and *b*) or different amounts of POPE added to PC liposomes that also contain 10% POPA (Fig. 7, *c* and *d*). From these data, we calculated the Michaelis constant *K_m_* and the turnover number *k*_cat_ using non-linear regression analysis (GraphPad Prism 6.0). For both GLUT3 and GLUT4, increasing concentrations of anionic or conical lipid increased the turnover number linearly but had no measurable effect on the Michaelis constant (Fig. 8). These data support a mechanism in which anionic and conical lipids increase the transporter interconversion rate and/or the fraction of active transporters without changing substrate binding affinity. Therefore, at saturating substrate concentration, anionic and conical lipids alter the rate-determining step in facilitative glucose transport. Similar to previous studies (34), the reconstituted transporter GLUT3 showed higher affinity for d-glucose than GLUT4, although it transported its substrate with a lower turnover number than GLUT4. The *K_m_* value for GLUT3 (11.9 ± 1.6 mm) and GLUT4 (33.3 ± 5.1 mm) reconstituted in liposomes was higher than reported when expressed in cells, which is consistent with previously published studies comparing transporter kinetics in cells *versus* reconstituted into liposomes (35–39). The *K_m_* (30.5 ± 2.1 mm) and *k*_cat_ (19.5 ± 0.57 s^−1^) values measured for GLUT4 in liposomes containing PC, PE, PI, PS, and PA (68.3, 16.7, 1.6, 10.6, and 2.8 mol %, respectively) were similar to those shown in Fig. 8. Measuring transporter kinetics of GLUT4 expressed in HEK293 cells prior to purification and reconstitution, we observed a lower *K_m_* of 7.1 ± 1.1 mm (Fig. 9), similar to previously published studies (34, 40). These results indicate that additional cellular components, not present in the purified four-component liposome system, might play a role in altering the transporter's *K_m_*. Although we cannot fully exclude the possibility that there are biophysical effects from membrane curvature in liposomes compared with conditions present in plasma membranes, we did not observe any differences in transport activity when measured in liposomes obtained from 200 nm *versus* 400 nm extrusion (data not shown).

The slope of the transporter *k*_cat_ plotted against the concentration of anionic or conical lipids correlated with the intensity of the lipid effect on transporter activity and allowed direct comparison of isoform-selective lipid effects. For both the anionic phospholipid PA and the conical lipid PE, the kinetic data show a 5-fold higher slope for GLUT4 compared with GLUT3, indicating a significantly stronger dependence of GLUT4 on anionic and conical lipids for activity. As expected, extrapolation to 0% PA indicated a negligible turnover number for GLUT4 where very little specific transport was observed. However, GLUT3 retained minimal activity even in the absence of anionic phospholipids. This correlates with our earlier data in which transport activity was measured in liposomes containing only PC or PC plus PE (Fig. 5*e*).

## Discussion

With a markedly expanding understanding of the biophysical effects of lipids on membrane proteins, advanced techniques for mammalian membrane protein purification, a growing list of crystal structures for transport proteins, and emerging methodologies to investigate protein conformational dynamics, it is imperative to systematically study the dependences of individual lipids on membrane protein activity. Taken together, our data demonstrate that anionic and conical lipids have major effects on the functional activity of the mammalian glucose transporters GLUT4 and GLUT3. However, the differences observed between these lipids suggest distinct mechanisms of transporter activation. A key finding is that anionic phospholipids are essential for glucose transport activity with this effect mediated through the lipid headgroup (Fig. 4*c*). All activating anionic phospholipids tested share the same fatty acids and a negative charge on the phosphate group but differ in the headgroup substituents (Fig. 4*e*). The zwitterionic lipids that were examined also share the same fatty acid moieties but carry a positive charge on their headgroup substituent that neutralizes the negatively charged phosphate group and do not activate the transporters (Fig. 4*a*). The anionic phospholipid POPA shows potent activation of the transporters but carries no headgroup substituent, suggesting that the negatively charged phosphate group mediates the activating effect as the only common denominator. The stabilizing effect of anionic phospholipids (Fig. 3) and the differential effect of various headgroup structures, carrying the same charge on GLUT4 protein (Fig. 4*c*), provide evidence for a direct interaction of annular anionic phospholipids with the transporter in contrast to an effect mediated through changes of the lipid bilayer chemical properties by bulk lipids. Hinging on the fact that anionic phospholipids are found exclusively on the inner leaflet of mammalian PMs, we propose that the interaction partners of the negative charge on the lipid are cationic residues on the cytoplasmic face of the transporters. The differences in GLUT4 and GLUT3 activation through anionic phospholipids with chemically different headgroup substituents suggest distinct interaction affinities of the different anionic lipids to the two transporter isoforms (Fig. 4, *c* and *d*).

Conversely, conical or non-bilayer lipids alone are not sufficient for GLUT4 or significant GLUT3 activity but increase transporter activity dramatically in the presence of anionic phospholipids (Fig. 5, *a* and *e*). Interestingly, this effect is not dependent upon the headgroup, charge, or acyl chains but is most likely mediated through their physical shape in the membrane. Lipid shape is determined by the ratio of headgroup to tail cross-sectional area. Conical lipids have large tails, often carrying single or multiple double bonds and small headgroups, whereas cylindrical lipids have tails and headgroups of similar size. The conical lipid POPE and the cylindrical lipid palmitoyloleoyl phosphatidylcholine, the main constituent of egg PC, share the same tail and charge and only differ in the methylation of the ethanolamine headgroup substituent (Fig. 4*e*). POPE is primarily found on the inner leaflet of mammalian PMs, affecting its biophysical properties by introducing lipid packing defects, curvature frustration, and altering the lateral pressure throughout the membrane (1, 3, 33). POPE increased transporter activity in a dose-dependent manner in the presence of anionic phospholipid (Fig. 5, *a* and *e*). Even more strikingly, the DAG lipid tested, which shares the same acyl chains as POPE but completely lacks the phosphate headgroup, making it even more conically shaped than POPE (Fig. 5*c*), showed a greater than 2-fold greater activation of GLUT4 compared with POPE (Fig. 5*d*). This ability of nonbilayer lipids to stimulate protein activity in the presence of anionic phospholipids has been demonstrated for several other integral membrane proteins (6, 29, 31).

Our results show that anionic and conical lipids influence the activity of GLUT4 and GLUT3 in different ways. Anionic phospholipids are necessary and sufficient for activity, whereas conical lipids alone are not required but increase activity significantly in the presence of anionic phospholipids. Additionally, the presence of anionic phospholipids increases the stability of GLUT4 as determined by SEC, whereas conical lipids have no influence on protein stability, indicating direct interaction of only anionic phospholipids to the transporter. Moreover, our kinetic analysis of both transporters revealed that anionic and conical lipids increase the *k*_cat_ linearly in a concentration-dependent manner, whereas the *K_m_* value remains unchanged. This suggests that both lipids do not change the affinity of the transporters for their substrate d-glucose but change either the turnover rate or the fraction of active transporters. We therefore propose the following working model for the anionic and conical lipid activation of the glucose transporters. According to recently published crystal structures of the GLUT isoforms GLUT1, GLUT3, and GLUT5 in inward- and outward-facing conformations (22–24), we assume that the transport of glucose is controlled by alternating between inward- and outward-facing conformations via a rocker-switch motion and by a gated-pore mechanism involving transmembrane helices 7 and 10. Alternation between inward- and outward-facing conformations is catalyzed by the equilibrium of formation and breaking of transient salt bridges at the cytoplasmic side of the transporter between the ends of several inter-transmembrane helices as well as between intracellular and transmembrane helices (22). We propose that anionic phospholipids interact with specific cationic amino acids, either the same residues involved in the salt bridges or unique ones, to stimulate the interconversion between inward and outward conformations, which is consistent with the observed anionic phospholipid concentration-dependent increase of *k*_cat_ (Fig. 8, *a* and *b*). Interestingly, a similar mechanism involving salt bridge-forming cationic amino acids that switch between Coulomb interactions with anionic amino acids and intermolecular interactions with anionic phospholipid headgroups has been proposed in a recent molecular dynamics simulation study of the β_2_-adrenergic receptor in lipid bilayers (41).

In contrast, conical lipids in the bilayer introduce lipid packing defects, alter the lateral pressure and membrane fluidity, and create curvature frustration thereby leading to increased flexibility and movement of integral membrane proteins (1, 3, 42, 43). Consequently, in the absence of conical lipids, the glucose transporters are embedded into a bilayer of mainly cylindrical lipids that provide tight packaging and create strong lateral pressure at the lipid-water interface, thus stabilizing the transporters' low energy conformations. In our working model, we propose that the increase in membrane flexibility by conical lipids leads to an accelerated rate of interconversion, consistent with the observed conical lipid concentration-dependent increase of *k*_cat_ (Fig. 8, *c* and *d*).

Although further work is required to determine the differences and similarities between GLUT4 and GLUT3 in detail, our findings establish that both transporters require anionic and conical lipids for optimal activity. Similar to the previously findings by Tefft *et al.* (21) for GLUT1 in liposomes composed of 100% anionic phospholipid, we determined that GLUT4 and GLUT3 show a strong headgroup preference among anionic phospholipids. Importantly, the observed effects of anionic phospholipid on GLUT4 and GLUT3 are observed at physiologically relevant lipid levels. GLUT4 is most active in the presence of PA (Fig. 4*c*), whereas GLUT3 and GLUT1 show highest activity with PS (Fig. 4*d*) (21). In contrast to GLUT4, GLUT3 and GLUT1 showed small but measurable glucose transport activity in PC liposomes in the absence of anionic phospholipids. Anionic phospholipid effects on transporter kinetics are consistent for all three transporters, affecting only the turnover number but not the *K_m_* value (21). The same kinetic observation was made for conical lipids for GLUT3 and GLUT4. The turnover number of GLUT4, however, was affected more strongly by anionic and conical lipids compared with GLUT3 (Fig. 8).

Although this study focused on phospholipid effects, native cell membranes are complex and contain other components that can influence glucose transporter function. Cholesterol in particular can constitute up to 50% of the plasma membrane composition in some tissues. Influences of cholesterol on GLUT1 activity have previously been reported (45, 46). These cholesterol effects may be mediated through changes in protein translocation or by direct effects on transporter activity. The latter could occur through direct cholesterol-protein interaction or via alteration of the biophysical properties of the lipid bilayer such as membrane thickness/hydrophobic matching, lateral pressure, or membrane fluidity (47, 48). Using the experimental approach that we have developed to precisely manipulate membrane composition, it will be possible to investigate the complex effects of cholesterol and other membrane constituents on the kinetic behavior of each of the mammalian GLUT isoforms.

The lipid sensitivity of GLUT4 and GLUT3 may have important biological implications. Mammalian lipid composition varies significantly in different tissues and also between cellular compartments (2, 49–52). Concurrently, both transporters show tissue-specific expression patterns with specialized functions in different cell types. Therefore, anionic and conical lipid concentrations in specific tissues or cellular compartments may coincide with transporter expression and their lipid requirements for activity. Particularly, the concentrations of the anionic lipids PS and PI as well as the conical lipid PE are highly dependent on the cellular organelle. The range of concentrations that we found to affect transporter function correlates with concentrations that differ between the endoplasmic reticulum, the Golgi apparatus, late endosomes, mitochondria, and the plasma membrane (2, 44), strongly suggesting a physiological role of anionic and conical lipid concentration in transporter activity within different cellular compartments. Interestingly, the maximal effect of conical lipids on GLUT4 activation (Fig. 5*a*) correlates with the physiological concentration of PE in mammalian PMs (Fig. 6*a*). At this concentration of conical lipids, anionic lipids further increased the activity of GLUT4 linearly beyond physiological concentrations, thereby opening an avenue for regulation of GLUT activity by pharmacological alteration of anionic lipid concentrations. Furthermore, pathological changes in lipid composition have been reported in the field of oncology, neurodegenerative disorders, and cardiovascular diseases (44). These changes could affect transporter function and thereby contribute to disease progression, again making lipid bilayer composition a promising pharmacological target. Considering the wide family of solute carriers implicated in human disease, GLUT4 and GLUT3 have significant sequence and structural homology to many transporters in this class. Therefore, it will be highly important to determine whether the lipid dependence reported herein is a general phenomenon for the entire class of SLCs or specific for certain transporters and what the effect the lipid activation has in the biological function of specific transport proteins.

## Experimental Procedures

### 

#### 

##### Materials

Egg PC, POPE, palmitoyloleoyl phosphatidylserine, POPA, palmitoyloleoyl phosphatidylglycerol, liver PI, brain SM, and 16:0–18:1 glycerol (DAG) were purchased from Avanti Polar Lipids Inc. (Alabaster, AL). LMNG and OMNG detergents were obtained from Anatrace (Maumee, OH). All other reagents were purchased from Sigma or as otherwise indicated.

##### GLUT4 Expression and Purification

Stable tetracycline-inducible HEK293S GnTI cell lines expressing GLUT4, tagged at the N and C termini with FLAG and His_9_, respectively, with a TEV protease cleavage site, were generated as described previously (28). Protein was purified as described in Ref. 28. Briefly, frozen cell pellets were thawed in a 30 °C water bath and resuspended in buffer B (50 mm potassium phosphate, 130 mm KCl, 15% (v/v) glycerol, 3 mm EDTA, protease inhibitor, 1 μg/ml DNase I (Roche Applied Science), and 1 mm PMSF (Fisher), pH 7.4) containing 0.75% (w/v) LMNG. Cells were broken using a 15-ml Dounce homogenizer and then rotated at 4 °C for 1 h. Cell lysate was spun at 75,000 × *g* for 45 min at 4 °C, and the supernatant was filtered through a 0.45-μm filter to remove insoluble matter. The filtered supernatant was incubated with washed M2 anti-FLAG affinity gel (Sigma) according to the manufacturer's instructions for 3 h with rotation at 4 °C. GLUT4-bound FLAG affinity gel was collected in a column and subjected to extensive washing with 30 column volumes of buffer C (50 mm potassium phosphate, 350 mm KCl, 15% (v/v) glycerol, 3 mm EDTA, complete protease inhibitor (Roche Applied Science), 0.1% (w/v) LMNG, pH 7.4). GLUT4 protein was eluted with six subsequent additions of 0.5 column volumes of elution buffer (buffer C containing 200 μg/ml FLAG peptide, 100 μg/ml 3× FLAG peptide (ApexBio, Houston, TX)). Tags were cleaved using TEV protease (plasmid purchased from Addgene, Cambridge, MA) at a ratio of 1:20 TEV/GLUT4 overnight at 4 °C in the presence of 1 mm DTT. Based on SDS-PAGE and Blue BANDit protein stain (Amresco, Solon, OH), GLUT4 protein after FLAG elution was >90% pure.

##### GLUT3 Expression and Purification

The gene for SLC2A3 (GLUT3) was purchased from the Mammalian Gene Collection IMAGE:4396508. GLUT3 (N43A) was expressed using a construct consisting of the full-length deglycosylation mutant SLC2A3 gene with a C-terminal purification tag containing a TEV protease cleavage site, a His_10_ purification sequence, and a FLAG tag in the expression vector pFB-CT10HF-LIC (available from the Structural Genomics Consortium). Baculoviruses were produced by transformation of DH10Bac cells. *Spodoptera frugiperda* (Sf9) insect cells in Sf-900 II SFM medium (Life Technologies, Inc.) were infected with recombinant baculovirus and incubated for 72 h at 27 °C in shaker flasks.

Cell pellets (4–6 liters were used for each purification) from 1 liter of insect cell culture were resuspended in 50 ml of lysis buffer (50 mm HEPES, pH 7.5, 200 mm NaCl, Roche Applied Science protease inhibitor mixture) and lysed by two passes through an EmulsiFlex-C5 homogenizer (Aventis, Ontario, Canada). Protein was extracted from cell membranes by incubation of the crude lysate with 1% octyl glucose neopentyl glycol (OGNG) and 0.1% cholesteryl hemisuccinate (CHS) for 1 h at 4 °C. Cell debris and unlysed cells were removed by centrifugation at 35,000 × *g* for 1 h. Detergent-solubilized protein was purified by immobilized metal affinity chromatography by batch binding to Co^2+^-charged TALON resin (Clontech) at 4 °C for 1 h. The resin was washed with 20 column volumes of wash buffer (50 mm HEPES, pH 7.5, 300 mm NaCl, 5% glycerol, 20 mm imidazole with 0.18% OGNG and 0.018% CHS) and eluted with lysis buffer supplemented with 250 mm imidazole. Imidazole was immediately removed using a PD10 column (GE Healthcare) and elution buffer lacking imidazole and glycerol. The PD10-eluted protein was treated with 20:1 (w/w, protein/protease) TEV protease overnight at 4 °C. The TEV protease-cleaved protein was separated from the His_6_-tagged TEV protease and uncleaved SLC2A3 by incubation for 1 h with TALON resin at 4 °C. The resin was collected in a column, and the flow-through and initial wash with SEC buffer (20 mm HEPES, pH 7.5, 100 mm NaCl, 0.12% OGNG, and 0.012% CHS) were concentrated in a 50-kDa cutoff, 2 ml of polyethersulfone concentrator (Corning), and further purified by SEC using a Superdex 200 10/300GL column (GE Healthcare) equilibrated with SEC buffer collected at 2 mg/ml. The molecular weight of each purified GLUT3 construct was confirmed using an MSD-ToF electrospray ionization orthogonal time-of-flight mass spectrometer (Agilent Technologies, Palo Alto, CA).

##### SEC Stability Study

FLAG-purified GLUT4 was injected onto a Superdex 200 10/300 GL size exclusion column (GE Healthcare) pre-equilibrated with 50 mm sodium phosphate, 150 mm NaCl, 0.1% LMNG, pH 7.4, to purify monomeric GLUT4. 40 μg of monomeric SEC-purified GLUT4 in 0.1% LMNG was then incubated in the absence and presence of various LMNG-solubilized lipids (0.02% w/v) for 1.5 h at 40 °C. Lipid-dependent stabilization from heat-induced destabilization of GLUT4 was monitored by SEC. All SEC experiments were conducted at a flow rate of 0.55 ml/min.

##### Liposome Reconstitution

GLUT4- and GLUT3-containing liposomes were assembled according to Kraft *et al.* (28). Briefly, lipids were mixed in chloroform and dried down under argon, and residual chloroform was removed under vacuum for 4 h. The dried lipid film was resuspended in buffer D (50 mm potassium phosphate, 130 mm NaCl, 10% (v/v) glycerol, complete protease inhibitor (Roche Applied Science), pH 7.4) with rigorous vortexing under argon. Lipids were hydrated for 1 h at room temperature using a vortexer set to the lowest speed followed by five freeze/thaw cycles in liquid nitrogen and 37 °C. The resulting multilamellar vesicles were extruded 11 times through 200-nm polycarbonate filters (Whatman, Florham Park, NJ) using a mini-extruder (Avanti Polar Lipids) according to the manufacturer's instructions to form large unilamellar vesicles. Triton X-100 destabilized liposomes were rotated with purified GLUT4 or GLUT3 (both with tags removed) at a lipid to protein ratio of 100–125:1 (w/w) for 45 min at 4 °C. Amberlite XAD-2 beads (Supelco Analytical, Bellefonte, PA) were added to remove detergent at a wet weight of 15.5 mg of beads per mg of Triton X-100, and samples were then rotated at 4 °C for 1 h. Fresh beads were added three additional times for 1 h, once more overnight, and one additional time for 2 h the following day. After removal of all beads, liposomes were collected by ultracentrifugation at 4 °C for 1 h at 267,000 × *g* and resuspended in buffer containing 50 mm potassium phosphate, pH 7.4, 130 mm KCl, pH 7.4, and complete protease inhibitor (Roche Applied Science). Samples were flash-frozen with liquid nitrogen before storing samples at −80 °C.

##### Liposome [^3^H]Glucose Uptake

Frozen liposomes containing purified GLUT4 or GLUT3 were thawed at room temperature and subjected to three freeze/thaw cycles (liquid nitrogen/room temperature) followed by extrusion through a 200-nm polycarbonate filter to create liposomes of uniform size distribution. Uptake was started by adding d-[^3^H]glucose (1 μCi/ml, 200 μm cold d-glucose) (American Radiolabeled Chemicals, St. Louis, MO) to GLUT4 or GLUT3 proteoliposomes at room temperature. Transport was stopped at different times with 5 ml of ice-cold quench buffer (50 mm potassium phosphate, pH 7.4, 130 mm KCl, pH 7.4, and 100 μm phloretin) and then filtered by vacuum on a 0.2-μm mixed cellulose ester filter (Advantec, Durham, NC). Filters were subsequently washed with an additional 10 ml of quench buffer. Scintillation fluid was added to the filters and the radioactivity counted. l[^3^H]Glucose (1 μCi/ml, 200 μm cold l-glucose) was used to determine nonspecific transport. Specific uptake was calculated for all experiments by subtracting nonspecific l-[^3^H]glucose from d-[^3^H]glucose uptake. In single time point experiments, specific uptake was normalized to the amount of transporter per reaction. The amount of transporter protein used in each uptake assay was determined by analyzing aliquots of transporter-containing liposomes along with a BSA standard curve on an SDS-polyacrylamide gel stained with Blue Bandit protein stain and quantified using an Odyssey infrared imaging system (LI-COR Biosciences, Lincoln, NB). Data were fitted by non-linear regression analysis using GraphPad Prism 6.0.

##### d[^3^H]Glucose Uptake in HEK293 Cells

Stably transfected tetracycline-inducible HEK293S GnTI cells expressing GLUT4 were grown on PEI-treated 12-well plates to 90% confluency for uptake experiments. 72 h prior, protein expression was induced by 2 μg/ml doxycycline hyclate and 5 mm sodium butyrate, although the control cells were left uninduced. Cells were glucose-starved in HEPES-buffered saline at room temperature 30 min prior to uptake measurements. Uptake was initiated in HEPES-buffered saline at room temperature with the addition of d-[^3^H]glucose and then quenched after 40 s by washing rapidly in ice-cold PBS. Intracellular radioactivity was quantified by liquid scintillation counting and normalized to total protein (BCA Protein Assay, Thermo Scientific, Rockford, IL). GLUT4-specific uptake was calculated by subtracting uptake from uninduced cells from that of induced cells.

## Author Contributions

R. C. H. and T. E. K. conceived and designed the research with oversight by P. W. H. Creation and optimization of the reconstitution and uptake protocol was performed by R. C. H. together with T. E. K. Experiments were performed and analyzed by R. C. H. and T. E. K. The SEC lipid stability assay and the GLUT4 expression, purification, and characterization system was conceived and developed by T. E. K. E. P. C. proposed the GLUT3 studies. Expression and purification of GLUT3 was performed by A. Q. and supervised by E. P. C. The paper was written by T. E. K., R. C. H. and P. W. H., with contributions from all co-authors. T. E. K. produced the figures.

## Supplementary Material

Supplemental Data

## Footnotes

6 The abbreviations used are:

PE: phosphatidylethanolamine
CHS: cholesteryl hemisuccinate
DAG: diacylglycerol
GLUT: glucose transporter
LMNG: lauryl maltose neopentyl glycol
OGNG: octyl glucose neopentyl glycol
PA: phosphatidic acid
PC: phosphatidylcholine
PI: phosphatidylinositol
PM: plasma membranes
PS: phosphatidylserine
POPA: palmitoyloleoyl phosphatidic acid
POPE: palmitoyloleoyl phosphatidylethanolamine
SEC: size exclusion chromatography
SM: sphingomyelin
SLCs: solute carriers
TEV: tobacco etch virus
ANOVA: analysis of variance.
