# Supplementary material for: Mammalian Glucose Transporter Activity Is Dependent upon Anionic and Conical Phospholipids
Source: J Biol Chem. 2016 Jun 14;291(33):17271–82. doi: 10.1074/jbc.M116.730168 (PMC5016126; doi:10.1074/jbc.M116.730168)
Supplement: Supplemental Data [file supp_291_33_17271__index.html]

Mammalian Glucose Transporter Activity is Dependent upon Anionic and Conical Phospholipids — Mammalian Glucose Transporter Activity Is Dependent upon Anionic and Conical Phospholipids — GLUT Activity Dependence on Phospholipids — Supplemental Data 

# Mammalian Glucose Transporter Activity Is Dependent upon Anionic and Conical Phospholipids

## Supplemental Data

- Supplemental Table 1 (.docx, 15 KB)
- Supplemental Table 2 (.docx, 15 KB)
