# Supplementary material for: Mammalian Glucose Transporter Activity Is Dependent upon Anionic and Conical Phospholipids
Source: J Biol Chem. 2016 Jun 14;291(33):17271–82. doi: 10.1074/jbc.M116.730168 (PMC5016126; doi:10.1074/jbc.M116.730168)
Supplement: Supplemental Data [file 10.1074_M116.730168_jbc.M116.730168-1.docx]

Supplementary Table 1. Total PM lipid composition (mole %)

|  | PC | +PS | +PE | +SM | +PI | +PA |
| --- | --- | --- | --- | --- | --- | --- |
| eggPC | 100 | 92 | 68 | 45 | 41.5 | 40.5 |
| POPS | 0 | 8 | 8 | 8 | 8 | 8 |
| POPE | 0 | 0 | 24 | 24 | 24 | 24 |
| SM | 0 | 0 | 0 | 23 | 23 | 23 |
| PI | 0 | 0 | 0 | 0 | 3.5 | 3.5 |
| POPA | 0 | 0 | 0 | 0 | 0 | 1 |
