# Supplementary material for: Mammalian Glucose Transporter Activity Is Dependent upon Anionic and Conical Phospholipids
Source: J Biol Chem. 2016 Jun 14;291(33):17271–82. doi: 10.1074/jbc.M116.730168 (PMC5016126; doi:10.1074/jbc.M116.730168)
Supplement: Supplemental Data [file 10.1074_M116.730168_jbc.M116.730168-2.docx]

Supplementary Table 2. PM inner leaflet lipid composition (mole %)

|  | PC | +PS | +PE | +SM | +PI | +PA |
| --- | --- | --- | --- | --- | --- | --- |
| eggPC | 100 | 84 | 47 | 33 | 26 | 24 |
| POPS | 0 | 16 | 16 | 16 | 16 | 16 |
| POPE | 0 | 0 | 37 | 37 | 37 | 37 |
| SM | 0 | 0 | 0 | 14 | 14 | 14 |
| PI | 0 | 0 | 0 | 0 | 7 | 7 |
| POPA | 0 | 0 | 0 | 0 | 0 | 2 |
